# Stromal-Cell-Derived Factor-1 (SDF-1)/CXCL12 as Potential Target of Therapeutic Angiogenesis in Critical Leg Ischaemia

**DOI:** 10.1155/2012/143209

**Published:** 2012-02-22

**Authors:** Teik K. Ho, X. Shiwen, D. Abraham, J. Tsui, D. Baker

**Affiliations:** ^1^Division of Surgery and Interventional Science, University College London (Royal Free Campus), The Royal Free Hospital, Pond Street, London NW3 2QG, UK; ^2^Centre for Rheumatology and Connective Tissue Disease, The Royal Free Hospital, Pond Street, London NW3 2QG, UK

## Abstract

In the Western world, peripheral vascular disease (PVD) has a high prevalence with high morbidity and mortality. In a large percentage of these patients, lower limb amputation is still required. Studies of ischaemic skeletal muscle disclosed evidence of endogenous angiogenesis and adaptive skeletal muscle metabolic changes in response to hypoxia. Chemokines are potent chemoattractant cytokines that regulate leukocyte trafficking in homeostatic and inflammatory processes. More than 50 different chemokines and 20 different chemokine receptors have been cloned. The chemokine stromal-cell-derived factor-1 (SDF-1 aka CXCL12) is a constitutively expressed and inducible chemokine that regulates multiple physiological processes, including embryonic development and organ homeostasis. The biologic effects of SDF-1 are mediated by chemokine receptor CXCR4, a 352 amino acid rhodopsin-like transmembrane-specific G protein-coupled receptor (GPCR). There is evidence that the administration of SDF-1 increases blood flow and perfusion via recruitment of endothelial progenitor cells (EPCs). This review will focus on the role of the SDF-1/CXCR4 system in the pathophysiology of PVD and discuss their potential as therapeutic targets for PVD.

## 1. Introduction

Atherosclerotic peripheral vascular disease (PVD) is a major cause of morbidity and mortality in the Western world [1]. It has been reported to have 19.1% prevalence in those over 55 years of age [2], and 15% of male patients die within 5 years of diagnosis, with the deaths mostly due to other associated atherosclerotic disease such as stroke, coronary heart disease, and abdominal vascular disease [3]. With an aging population and improved medical care that has increased life expectancy, more patients are presenting with critical leg ischemia (CLI), the end stage of PVD. In 10 to 40% of these patients [4], lower limb amputation may be required because the anatomic extent and the distribution of arterial occlusive disease make the patients unsuitable for revascularization, despite current advances in surgery and endovascular revascularization techniques.

In the past 20 years, rapid development in molecular biology and understanding of mechanisms of angiogenesis [5] have led to the development of therapeutic angiogenesis as a promising strategy to treat a variety of cardiovascular diseases. Experimental animal model studies [6] have progressed to several clinical trials which evaluated the angiogenic potentials of growth factors such as hepatocyte growth factor (HGF), vascular endothelial growth factor (VEGF), and fibroblast growth factor (FGF) in inducing new blood vessel growth in CLI patients. Unfortunately, the results of these clinical trials have been inconsistent and inconclusive. For example, in the regional angiogenesis with VEGF (RAVE) trial [7], intramuscular adenoviral delivery of VEGF in patients with unilateral exercise-limiting claudication had no effects on primary efficacy end points but was associated with dose-dependent peripheral oedema. VEGF induces formation of hyperpermeable and proinflammatory vessels; the concerted actions of additional mediators (such as the angiopoietins and platelet-derived growth factor) may be required for their stabilization and maturation. This view has been supported by the use of a combination of two angiogenic factors, platelet-derived growth factor BB and FGF-2, which synergistically induce vascular networks that remain stable for more than a year even after depletion of angiogenic factors [8].

Chemokines are potent chemoattractant cytokines that regulate leukocyte trafficking in homeostatic and inflammatory processes. More than 50 different chemokines and 20 different chemokine receptors have been cloned [9]. Chemokines usually bind to multiple receptors, and the same receptor may bind to more than one chemokine. However, there is one exception to this rule: SDF-1, which binds exclusively to CXCR4, and has CXCR4 as its only receptor [9–16]. Recently, a new putative receptor called CXCR7 (aka RDC1 and GPR159) for SDF-1 had been described [17]; however, its potential role in regulating cell trafficking awaits confirmation by other laboratories.

This review will focus on the role of stromal-cell-derived factor- (SDF-1), otherwise known as CXCL12, and its receptor CXC chemokine receptor 4 (CXCR4; i.e., the SDF-1/CXCR4 system) in the pathophysiology of PVD and discuss their potential as therapeutic targets for PVD.

## 2. Stromal-Cell-Derived Factor- (SDF-1)

The chemokine stromal-cell-derived factor-1 (SDF-1, also known as CXCL12) is a constitutively expressed and inducible chemokine that regulates multiple physiological processes, including embryonic development and organ homeostasis [18]. SDF-1 is the only member of the *α*-chemokines which does not possess the conserved Glu-Leu-Arg motif, also called the ELR motif, preceding the first cysteine residue but has angiogenic activity. Two main splice forms of SDF-1 have been identified, SDF-1*α* and SDF-1*β*, which have identical amino acid sequences except for the presence of 4 additional amino acids at the carboxy terminus of SDF-1*β* [19]. Another splice variant form, SDF-1*γ*, has been subsequently identified in the nervous system [20]. SDF-1*δ*, SDF-1*ε*, and SDF-1*φ* splice variants have been described only recently in human tissues and are abundant in the pancreas. Additionally, SDF-1*φ* and SDF-1*ε* are found in the heart and liver, as well as in fetal and adult kidneys, but the expression of SDF-1*φ* is more pronounced than SDF-1*ε*. SDF-1*δ*, on the other hand, is also detected in the spleen, fetal liver, and lungs [21].

SDF-1*α* is the predominant isoform found in all organs but undergoes rapid proteolysis in blood. SDF-1*β* is more resistant to blood-dependent degradation, stimulates angiogenesis, and is present in highly vascularized organs such as the liver, spleen, and kidneys. In contrast, SDF-1*γ* is located in very metabolically active organs susceptible to infarction such as the heart and the brain. The significance of the existence of the splice forms of SDF-1 has largely remained unclear. The understanding of the functional diversity of the different splicing variants will help in developing therapeutic strategies. Structure-function analysis of SDF-1*α* (1–67) has identified the NH_2_-terminal amino acids (residues 1–8) as critical to receptor binding and activation. The expression of SDF-1 in several organs including liver, brain, kidney, and heart increased in the presence of ischaemia [22]. Recently, it has been shown that SDF-1 expression in skeletal muscle is also elevated in ischaemic skeletal muscle fibres of patients with critical leg ischaemia [23]. Studies at the molecular level show that the SDF-1 promoter contains hypoxia inducible factor-1*α* (HIF-1*α*) binding sites, as revealed by chromatin immunoprecipitation analysis, and SDF-1 expression is upregulated in endothelial cells by HIF-1*α* [24]. Thus, it is possible that elevated levels of HIF-1*α* in critical leg ischaemia [25] may have led to increased SDF-1 in the ischaemic skeletal muscle fibres.

## 3. CXCR4

The biologic effects of SDF-1 are mediated by the chemokine receptor CXCR4. CXCR4 (aka fusin and CD184), is a 352 amino acid rhodopsin-like transmembrane specific G protein-coupled receptor (GPCR). CXCR4 is expressed by various cell lines, including muscle cell lines, endothelial cells, leucocytes, and progenitor cells [26–28] and the expression of CXCR4 receptor on endothelial cells has been reported to be regulated by hypoxia [29, 30] at transcriptional level. The regulation of CXCR4 can also occur during protein expression when it is subjected to a number of cotranslational modifications. These processes affect the expression and function of the receptor [31]. After expression, CXCR4 is exposed to proteolytic degradation by proteases that are present in the haematopoietic microenvironment and serum (e.g., cathepsin G, elastase, dipeptidyl-peptidase) [32, 33]. CXCR4 after interaction with SDF-1 is internalized from the surface in a mechanism involving G-protein-coupled receptor kinases followed by the binding of *β*-arrestin [34], and subsequently, is recirculated from the endosomal compartment at different rates: this achieves another level of regulation. The function of the CXCR4 receptor depends on its incorporation into membrane lipid domains called lipid rafts and several signals from other membrane receptors (e.g., G-protein-coupled C3a anaphylatoxin receptor—C3aR) or integrins that may increase the incorporation of CXCR4 into membrane lipid rafts, enhancing its signalling [35, 36]. Finally, CXCR4 is the subject of negative regulation by regulators of G-protein signalling (RGS) proteins which may regulate CXCR4 signalling differently in different tissues [37, 38].

## 4. SDF-1 CXCR4 Signalling Axis

Upon activation of CXCR4, a number of signalling pathways are activated leading to a variety of biological responses. As CXCR4 couples to the G_i_ family of proteins, pertussis toxin (PTX), which ADP-ribosylates G*α*_i_ and inhibits GPCR/G_i_ coupling, has been used to delineate pathways that are G-protein-dependent and -independent. Strong evidence exists that the protein-tyrosine phosphatases SHIP1 and SHIP2, as well as membrane-expressed hematopoietic phosphatase CD45, are involved in the modulation of CXCR4 signalling [39]. CXCR4 signalling is negatively regulated by the RGS proteins [37, 38] and lipid phosphatase activity of tumor suppressor phosphatase and tensin homolog (PTEN) [40].

### 4.1. G Protein Signalling

To date, the majority of signalling pathways and biological outcomes of CXCR4 activation are PTX sensitive and, therefore, dependent on activation of G_i_ proteins. Activated G_i_ is able to inhibit adenylyl cyclase as well as activate the Src family of tyrosine kinases while liberated G*βγ* activates phospholipase C-*β* (PLC-*β*) [31], phosphoinositide-3 kinase (PI3K) [41], and mitogen activated protein kinase (MAPK) [42] signalling pathways. As described earlier, the CXCR4/SDF-1 complex is incorporated into lipid rafts and may associate with members of the Src family of kinases [43]. Activated CXCR4 increases intracellular calcium mobilization and induces phosphorylation of focal adhesion components such as FAK and Pyk2 [44–46]. The PI3K/AKT and MAPK signal transduction pathways contribute to chemotaxis, cell migration [42, 47], and secretion of various matrix metalloproteinases (MMP's) including MMP-2 and MMP-9 [46, 48]. MMP-2 and MMP-9 are involved in the migration of cells through the basement membrane [49]. They also induce secretion of the angiopoietic factor, VEGF [50]. Furthermore, blocking either the PI3K or MAPK pathway inhibits CXCR4-activated cell migration in a pre-B cell line [42]. Ultimately, through these pathways, SDF1-bound CXCR4 can induce cell proliferation, chemotaxis, migration, the secretion of angiopoietic factors, all important components of the angiogenic process [51].

### 4.2. G Protein Independent Signalling

Activation of the JAK/STAT pathway by CXCR4 has been proposed to be G protein independent [52]. SDF-1 induces the transient association of JAK2 and JAK3 with CXCR4, leading to the activation and nuclear translocation of a number of STAT proteins. While JAK/STAT activation is G protein-independent, pretreatment with PTX leads to a prolonged association of JAK with CXCR4 suggesting that G protein coupling is involved in JAK/STAT-receptor complex recycling [52].

## 5. SDF-1, Vasculogenesis, and Angiogenesis

Until recently, new blood vessel growth in the adult was thought to occur exclusively through angiogenesis, defined as the sprouting of vessels from existing vascular structures. In contrast to angiogenesis, endothelial progenitor cells (EPCs) are mobilised from the bone marrow and recruited to foci of neovascularization where they form new blood vessels *in situ* through a process called vasculogenesis. Once thought to be limited to embryonic development, vasculogenesis appears to be preserved in the adult and contributes to postischemic vascular regeneration.

### 5.1. The Effect of SDF-1 and Endothelial Progenitor Cell (EPC) Trafficking on Vasculogenesis

In 1997, Asahara et al. reported the isolation of putative EPC from human peripheral blood, on the basis of cell-surface expression of CD34 and other endothelial markers [53]. These cells are reported to differentiate *in vitro* into endothelial cells and seem to be incorporated at sites of active angiogenesis in various animal models of ischaemia. These findings suggest that incorporation of bone-marrow-derived endothelial precursor cells into the new vessels lumen contributes to the growing vessels and complements the resident endothelial cells in sprouting new vessels. Also, ischaemia and various cytokines, including VEGF and granulocyte-macrophage colony-stimulating factor (GM-CSF), are reported to mobilize EPC into sites of neovascularization [54]. EPCs express CXCR4 which allows homing to sites of neovascularization in ischaemic tissues which release the homing signal, that is, SDF-1, which then act as ligand for CXCR4. Other CXCR4+ proangiogenic cells are composed of immature and mature hematopoietic cells, and smooth muscle cell (SMC) progenitors, which all have direct or indirect proangiogenic properties.

 SDF-1 is upregulated in ischaemic tissues as hypoxic and/or apoptotic conditions are triggers to the induction of cytokine and chemokine expression. Ceradini et al. have demonstrated that SDF-1 expression in ischaemic sites is directly correlated with the amplitude of hypoxia [24]. Signalling activated by hypoxia leading to SDF-1 upregulation involves the recruitment of integrin-linked kinase and HIF-1 [24, 55]. Acute and gradual arterial occlusion can both lead to increased SDF-1 expression in ischaemic limb [56]. However, the expression of SDF-1 can be affected by age, with decreased expression in older age [57]. We investigated the expression of two main splice variant, SDF-1*α* and SDF-1*β* in ischaemic human skeletal muscle of patients with critical leg ischaemia [23]. The study confirms the elevated expression of SDF-1*α* but there is lack of SDF-1*β* expression in ischaemic human skeletal muscle. It is postulated that the lack of SDF-1*β* expression in critical leg ischaemia may be explained by its transient expression [58] because all the muscles examined have been chronically ischaemic. Another human study [59] shows that SDF-1 expression increases in acute on chronic ischaemia with perivascular retention of CXCR4+ cells.

The increased expression of SDF-1 in ischaemic muscles acts as a chemoattractant and homing signal for CXCR4+ EPCs. Locally administered SDF-1 has been shown to augment the accumulation of EPCs to the site of ischaemia, resulting in enhancing the efficacy of neovascularization after systemic EPC transplantation [60]. The study provided experimental proof of principle for the feasibility and therapeutic effectiveness of local administration of SDF-1 in augmenting the SDF-1/CXCR4+ EPCs enhanced neovascularization in ischaemic muscles.

### 5.2. Angiogenic Properties of SDF-1

Many critical cell functions such as migration, proliferation, and apoptosis inhibition are regulated by SDF-1. There are limited studies in the literature investigating human microvascular endothelial cells which are better representatives of the microvasculature involved in angiogenesis. In a study comparing the angiogenic properties of both SDF-1*α* and SDF-1*β* splice variants [23], both splice variants attenuate human microvascular endothelial cells apoptosis and stimulate cell proliferation and capillary tube formation in a Matrigel assay. The Matrigel assay mimics various steps in angiogenesis such as proliferation, migration, and differentiation in the process of capillary tube formation. Compared with SDF-1*α*, SDF-1*β* has a greater effect on apoptosis and cell proliferation. Treatment with both variants results in time-dependent activation of PI3K/Akt and MAPK p44/42 but not p38 MAP kinase [23]. These properties are likely to contribute to the complex mechanism of angiogenesis.

## 6. Potential Use of SDF-1 as Therapeutic Angiogenic Factor

The ideal mode of delivery of angiogenic growth factors has yet to be determined. They are usually delivered by gene transfer, which commonly uses adenoviruses (Ads) and plasmids as vectors or delivered as recombinant protein. Each method has its own disadvantages [6] and the delivery of SDF-1 is no exception. Recently, two promising studies attempt to provide successful modes of SDF-1 delivery in ischaemic limbs to improve revascularisation using vascular gene transfer of SDF-1 by ultrasound-mediated destruction of plasmid bearing microbubbles and mutated recombinant SDF-1 proteins [61, 62].

The success of EPCs therapy relies on the ability of cells to repair damaged tissue, and it is critically dependent on the homing, migration, and retention to sites of ischaemia, regardless of mode of delivery. In an animal model of chronic ischaemic limb, it has been demonstrated that a noninvasive gene- and cell-based therapeutic approach, using vascular gene transfer of SDF-1 by ultrasound-mediated destruction of plasmid bearing microbubbles to augment homing and engraftment of exogenously administered EPCs, leads to a greater angiogenic response as compared to SDF-1 gene therapy or intravenous EPCs alone [61]. However, long-term data on EPC engraftment, beyond 14 days after delivery, are not available.

The use of SDF-1 protein therapy to enhance angiogenesis is hampered by concerns regarding rapid inactivation of the chemokine in the protease-rich environment of the ischaemic limb. SDF-1 processing by matrix metalloproteinase-2 results in generation of a neurotoxic fragment that does not bind to its main receptor, CXCR4, and lacks chemotactic activity for EPCs [63, 64]. To ensure sustained SDF-1 activity in the hostile environment of the ischaemic limb, Segers et al. [62] designed recombinant SDF-1 proteins carrying mutations that provide resistance to protease cleavage. One of these SDF-1 variants, SSDF-1(S4V), is resistant to processing by matrix metalloproteinase-2 and dipeptidyl peptidase IV but retains chemotactic activity *in vitro* and induced angiogenesis *in vivo*. Delivery of protease-resistant SDF-1 with the use of self-assembling nanofibres to achieve sustained local concentrations increases arteriolar density and enhances blood flow in the ischaemic mouse hindlimb.

Similar to treatment with the angiogenic growth factors basic fibroblast growth factor (FGF) and VEGF, administration of protease-resistant SDF-1 augments perfusion, increasing vascular density in the ischaemic limb. Considering the established effects of VEGF and basic FGF administration in enhancing experimental ischaemic angiogenesis, what additional role could SDF-1 treatment play? Compared with the effects of other angiogenic growth factors, SDF-1 has unique properties. Generation of hyperpermeable vessels, a major characteristic of VEGF-stimulated angiogenesis, may not be observed after injection of SDF-1. SDF-1 contributes to the stabilization of neovessel formation by recruiting CXCR4+PDGFR+ckit+ smooth muscle progenitor cells during recovery from vascular injury [65]. However, the angiogenic pathways involving VEGF and SDF-1 are not independent. Extensive evidence suggests that SDF-1 upregulates VEGF synthesis in several different cell types, whereas VEGF and basic FGF induce SDF-1 and its receptor CXCR4 in endothelial cells [66]. In a transgenic system of VEGF-mediated neovascularization, SDF-1 is a key mediator of retention of recruited bone-marrow-derived cells in close proximity to angiogenic vessels [67]. These interactions provide a link between angiogenic growth factors and chemokine-induced angiogenesis.

Although promising, the use SDF-1 in a clinical setting is uncertain and yet to be tested. The animals used for experimentation are typically young and healthy and have the same genetic background. In contrast, patients with PVD are a highly heterogeneous group comprising older individuals with enormous genetic diversity and various comorbid conditions, such as diabetes mellitus, hypertension, and hyperlipidemia.

## 7. Future Work

Better understanding of the mechanisms of action of the chemokine in the ischaemic limb is necessary for optimal exploitation of the SDF-1/CXCR4 axis in PVD. As discussed, SDF-1 not only exerts its effect on vasculogenesis by the recruitment of CXCR4+ progenitor cells, but it also has direct angiogenic properties on endothelial cells. The mechanism of actions causing neovascularisation in ischaemic limb is uncertain: one or both of the processes may be involved. In addition, there is uncertainty on the role and interaction of VEGF and other angiogenic growth factors with SDF-1 in the microenvironment of ischaemic limbs. The answers to these issues are paramount to understanding the interplay between various mediators of angiogenesis and to design therapeutic strategies combining protein and cell therapy approaches.

Moreover, the effects of SDF-1 on the inflammatory response in the ischaemic limb need to be understood. The roles of elevated expression of SDF-1 and its interaction with other angiogenic growth factors in ischaemic muscles need to be determined. There has been no study on how the increased expression of SDF-1 by ischaemic muscles affect serum levels of SDF-1, which can then affect the mobilisation of progenitor cells from bone marrow. Evidence from *in vivo *studies in models of inflammatory injury is conflicting, suggesting that SDF-1 exerts context-dependent proinflammatory and antiinflammatory actions [68].

The role of SDF-1 in establishing long-term stable and mature blood vessels remains unknown. A major limiting factor in angiogenic therapies is the formation of unstable vessels that may rapidly regress after cessation of treatment [69]. Whether SDF-1 mediates coating of the neovessels with pericytes and vascular maturation is not known. Formation of viable and stable neovessels *in vivo* may require the concerted effort of several distinct mediators. Finally, much work is needed to investigate the role of the newly discovered SDF-1 receptor CXCR7 on SDF-1 angiogenic properties.

To apply SDF-1 treatment in patients with PVD, certain issues will need to be considered, such as the effect of SDF-1 on atherosclerosis. Additional experiments using atherosclerotic animal models may shed light on this concern. Nevertheless, we believe that the concept of augmenting local accumulation of transplanted EPCs opens perspectives for the clinical strategy of EPC therapies.
